# Gender, FT4 levels, T stage, and BMI as predictors of TSH levels in thyroid cancer patients

**DOI:** 10.3389/fendo.2025.1422464

**Published:** 2025-01-24

**Authors:** Sen Zhang, Shuli Niu, Ling Zhou

**Affiliations:** Department of Nuclear Medicine, Deyang People’s Hospital, Deyang, Sichuan, China

**Keywords:** thyroid cancer, nomogram, thyrotropin suppression, individualized medication regimens, levothyroxine

## Abstract

**Background:**

After initial treatment, levothyroxine (LT4) administration is necessary for thyroid cancer patients to achieve target thyroid-stimulating hormone (TSH) levels. However, the clinical efficacy of weight-based LT4 dosing has been suboptimal, highlighting the need to identify factors influencing the attainment of desired TSH levels and guide personalized treatment.

**Methods:**

We constructed a retrospective cohort comprising 215 patients diagnosed with thyroid cancer. The identification of factors influencing the attainment of expected TSH levels was accomplished through univariate and multivariate logistic regression analyses. Subsequently, we developed a nomogram based on these prognostic factors and performed internal validation using the bootstrap resampling method.

**Results:**

Univariate and multivariate logistic regression analyses were conducted to analyze the clinical and demographic parameters. A nomogram was constructed using bootstrap resampling to predict the risk of TSH suppression failure, which was validated. The nomogram demonstrated moderate discrimination in estimating the risk of TSH suppression failure, with a Hosmer-Lemeshow test p-value of 0.393 and a bootstrapped calibrated C-index of 0.757 (95% CI 0.687-0.814). The calibration curve indicated good consistency of the model, and decision curve analysis suggested that the nomogram had clinical utility.

**Conclusion:**

Gender, preoperative serum free thyroxine (FT4) levels, T stage, and body mass index exhibit independent associations with the expected level of TSH. The established nomogram effectively predicts the risk of TSH suppression failure. Further research is warranted to investigate how these factors can be utilized in developing a personalized LT4 dosage calculator.

## 1 Introduction

Thyroid cancer is one of the most common types in the endocrine system, with more than 90% of newly diagnosed cases each year being differentiated thyroid cancer (1) (DTC). After thyroidectomy or subtotal thyroidectomy, and then with the management of radioiodine therapy, the 5-year survival rate for DTC is 90% or higher. Following total thyroidectomy, levothyroxine (LT4) is administered to compensate for the loss of thyroid function. However, intermediate- or high-risk patients have a recurrence risk of 5-20%, or even higher. To reduce this risk, supra-physiological doses of LT4 can be used to suppress the secretion of thyroid-stimulating hormone (TSH) (2), further lowering the risk of recurrence.

In clinical practice, the initial dosage of TSH suppression therapy is typically determined based on the patient’s weight, usually ranging from 1.6 to 2.2 μg/kg/day. However, this approach often lacks accuracy, as only approximately 40% of patients do not require dose adjustment during their first postoperative follow-up vistit (3). The remaining patients necessitate a period of time to identify the optimal LT4 dosage, which may extend beyond three months. Throughout this duration, patients not only endure adverse effects resulting from inadequate or excessive LT4 dosages but also encounter increased outpatient visits and laboratory tests that contribute to their financial burden.

In addition, estimating LT4 dosage based on body weight may result in excessive intake for overweight patients. When TSH is suppressed to below the lower limit of normal for a long period, it may bring adverse effects, manifested in the cardiovascular system and bone of postmenopausal women (4–7). To balance benefits and risks, there is a growing focus on personalized treatment, however, the differences in response to LT4 among individuals remain unresolved. The dosage of LT4 may be influenced by many factors. It has been shown that body mass index (BMI) and body surface area can effectively predict the initial dose of LT4 (8, 9). Sex, age, and body weight have been suggested may have an impact on the requirement for LT4 dosage (10, 11). Recently, a study suggests that preoperative TSH and serum free triiodothyronine (FT3) levels may be important factors affecting LT4 dosage (12). Given the multitude of factors influencing LT4 requirements, it is imperative to develop a predictive model that can accurately estimate LT4 dosages for diverse patient populations based on TSH compliance.

Based on the existing research findings, various demographic characteristics and/or thyroid function indicators can be utilized to predict TSH inhibition in patients with differentiated thyroid cancer. A nomogram is a dependable tool for constructing a simple and intuitive visualization of quantitative clinical prediction models (13). This study aims to identify the combination of variables affecting TSH compliance in patients with differentiated thyroid cancer and to construct a nomogram that effectively identifies high-risk populations with TSH non-compliance.

## 2 Materials and methods

### 2.1 Patients

This present retrospective study was approved by the medical ethics committee of Deyang People’s Hospital (2023-04-075-K01). Considering its retrospective nature, informed consent from all patients was waived. The clinical date of patients treated in the Department of Nuclear Medicine of Deyang People’s Hospital from January 2019 to December 2022 were reviewed, and patients meeting the following conditions were included in the study: (1) Age > 18 years old; (2) Pathologically confirmed differentiated thyroid cancer; (3) Complete clinical data; and (4) No preoperative use of thyroid hormone or iodine agent. Patients with suboptimal medication adherence and inconsistent follow-up visits were exclude from the study.

A total of 215 patients with histologically confirmed DTC were included in this study. Prior to surgery and radioiodine therapy (RAI), all patients underwent a comprehensive evaluation including physical examination, assessment of thyroid function, detection of thyroid peroxidase antibody (TPOAB) and thyroglobulin antibody (TgAB) levels. Additionally, TSH levels were measured 6 weeks after RAI. All detections were performed by electrochemiluminescence immunoassay and completed in the same laboratory, the reference range of TSH, FT3, serum free thyroxine (FT4) was 0.27~4.2mU/L, 3.1~6.8pmmol/L, 12~22pmol/L respectively. At the same time, we included the number of metastatic lymph, total number of lymph nodes removed, and lymph node metastasis rate as variables in the analysis.

The baseline characteristics of patients were obtained from the electronic medical record system, including age, gender, BMI, pathological T stage and N stage. Pathological staging referred to the 8^th^ edition (14) of TNM staging jointly formulate by the American Joint Committee on Cancer (AJCC) and the Union for International Cancer Control (UICC). The initial dosage of LT4 for TSH suppression therapy was 2.0μg/kg/day, administered as a single oral dose 30-60 minutes prior to breakfast.

### 2.2 Statistical analysis

All statistical analyses and data visualizations were performed using the R statistical software, version 4.3.2 (R Foundation for statistical Computing; statistical https://www.r-project.org/). Continuous variables were expressed as mean ± standard, categorical variables are expressed as percentages. Univariate logistic regression was used to evaluate all variables in the cohort to determine independent factors affecting TSH suppression. Variables with P < 0.05 were included in multivariable logistic regression analysis. Multiple models were built, and the one with the smallest AIC was chosen, followed by backward stepwise selection. Based on the final multivariate logistic regression analysis results, a nomogram was constructed to predict the effect of TSH suppression using R software.

The calibration curve was drawn to calibrate the collinearity plot, and the discrimination ability was evaluated by C-index. In addition, the collinearity plot was internally validated by 500 bootstrap resampling to evaluate its accuracy of prediction. The clinical net benefit of the model was evaluated by decision curve analysis.

## 3 Results

### 3.1 Patients characteristics

From January 2019 to December 2020, a total of 322 patients underwent total thyroidectomy and received RAI therapy. After applying the inclusion and exclusion criteria, there were a total of 215 patients with differentiated thyroid cancer who had complete baseline and laboratory data, as shown in **Table 1**. Among these patients, there 50 males (23.3%) and 165 females (76.7%), ranging in age from 19 to 78 years. At the week 6 follow-up, TSH suppression levels remained unachieved in a majority of patients (59.1%). The observation is noteworthy that, in comparison to males, females appear to encounter challenges in attaining the target of TSH suppression. Patients with lower T stage, lower BMI, lower lymph node metastasis rate, and lower preoperative FT4 and FT3 seemed to be more likely to achieve suppression goals.

**Table 1:** Clinical characteristics of patients.

Characteristics	Total n/mean(SD)	Expected level n(%)/mean ± SD	Unexpected level n(%)/mean ± SD
Number	215	127(59.1%)	88(40.9%)
sex:			
male	50 (23.3%)	43 (33.9%)	7 (7.95%)
female	165 (76.7%)	84 (66.1%)	81 (92.0%)
age	43.03 ± 12.35	42.35 ± 11.79	44.01 ± 13.12
BMI	23.58 ± 3.50	24.18 ± 3.43	22.71 ± 3.43
T stage:			
T1~T2	137 (63.7%)	70 (55.1%)	67 (76.1%)
T3 ~ T4	78 (36.3%)	57 (44.9%)	21 (23.9%)
N stage:			
No	19 (8.84%)	9 (7.09%)	10 (11.4%)
Yes	196 (91.2%)	118 (92.9%)	78 (88.6%)
recurrent risk:			
Low	33 (15.3%)	14 (11.0%)	19 (21.6%)
intermediate	104 (48.4%)	58 (45.7%)	46 (52.3%)
high	78 (36.3%)	55 (43.3%)	23 (26.1%)
preoperative TSH	2.99 ± 2.40	2.74 ± 1.81	3.35 ± 3.04
preoperative FT4	16.58 ± 2.84	17.10 ± 2.88	15.82 ± 2.64
preoperative FT3	4.94 ± 0.76	5.05 ± 4.79	4.79 ± 0.71
preoperative Tg	68.91 ± 126.63	81.14 ± 142.24	51.27 ± 98.03
preoperative TGAB	237.88 ± 723.97	220.49 ± 741.14	262.97 ± 701.87
preoperative TPOAB	61.58 ± 123.64	70.43 ± 140.74	48.82 ± 92.92
pre-ablation stimulated FT4	2.44 ± 1.29	2.39 ± 1.35	2.51 ± 1.19
pre-ablation stimulated FT3	1.21 ± 0.65	1.21 ± 0.72	1.21 ± 0.53
pre-ablation stimulated Tg	16.29 ± 58.22	13.63 ± 42.29	20.12 ± 75.65
pre-ablation stimulated TgAb	157.94 ± 543.90	147.62 ± 529.38	172.82 ± 566.94
preoperative FT4/FT3	3.40 ± 0.67	3.45 ± 0.72	3.34 ± 0.58
pre-ablation stimulated FT4/FT3	2.15 ± 0.79	2.17 ± 0.91	2.11 ± 0.57
Number of metastatic lymph nodes	6.01 ± 6.04	6.35 ± 5.92	5.53 ± 6.23
Number of lymph nodes removed	19.87 ± 14.98	19.65 ± 14.43	20.20 ± 15.82
Lymph node metastasis rate	0.33 ± 0.25	0.36 ± 0.25	0.29 ± 0.25
Smoking			
Yes	26 (12.1%)	20 (15.7%)	6 (6.8%)
No	189 (87.9)	107 (84.3%)	82 (93.2%)
Alcohol			
Yes	44 (20.5%)	29 (22.8%)	15 (17.0%)
No	171 (79.5%)	98 (77.2%)	73 (83.0%)
Hypertension			
Yes	23 (10.7%)	15 (11.8%)	8 (9.1%)
No	192 (89.3%)	112 (88.2%)	80 (90.9%)
Diabetes			
Yes	10 (4.7%)	4 (3.1%)	6 (6.8%)
No	205 (95.3%)	123 (96.9%)	82 (93.2%)

BMI, body mass index; TSH, thyroid-stimulating hormone; FT4, serum free thyroxine; FT3, serum free triiodothyronine; Tg, thyroglobulin; TGAB, thyroglobulin antibody; TPOAB, thyroid peroxidase antibody.

### 3.2 Model prediction factor selection

Utilizing the results from the univariate logistic regression analysis (**Table 2**), variables including sex, BMI, T stage, preoperative FT4, and lymph node metastasis rate were integrated into the multivariate logistic regression analysis. Employing a backward stepwise regression approach, the multivariate logistic regression analysis identified sex (P=0.003), BMI (P=0.048), T stage (P=0.001), and preoperative FT4 (P=0.008) as independent predictive factors for achieving the desired TSH suppression level. The analysis highlighted that male patients, particularly those with elevated preoperative FT4 levels, advanced T stage, and increased BMI, faced grater challenges in attaining the targeted TSH suppression while receiving a dosage of LT4 at 2.0μg/kg.

**Table 2:** Univariate and multivariate logistic regression analysis of clinical candidate predictors.

Variables	Univariate analysis			Multivariate analysis		
	OR	95% CI	P	OR	95% CI	P
sex	0.17	0.072-0.397	<0.01	0.24	0.1-0.61	0.003
age	0.99	0.968-1.011	0.333			
BMI	1.14	1.044-1.235	0.003	1.1	1-1.2	0.048
T stage	2.6	1.423-4.741	0.002	2.96	1.53-5.27	0.001
N stage	1.68	0.654-4.324	0.281			
recurrent risk	1.71	0.775-3.777	0.183			
preoperative TSH	0.9	0.795-1.013	0.079			
preoperative FT4	1.2	1.069-1.337	0.002	1.18	1.04-1.34	0.008
preoperative FT3	1.62	1.094-2.386	0.016			
preoperative Tg	1	1.000-1.004	0.096			
preoperative TGAB	1	1.000-1.000	0.672			
preoperative TPOAB	0.998	0.996-1.001	0.214			
pre-ablation stimulated FT4	0.93	0.752-1.148	0.495			
pre-ablation stimulated FT3	0.97	0.641-1.476	0.896			
pre-ablation stimulated Tg	1	0.994-1.002	0.431			
pre-ablation stimulated TgAb	1	1.000-1.001	0.739			
preoperative FT4/FT3	1.3	0.848-1.985	0.231			
pre-ablation stimulated FT4/FT3	1.1	0.773-1.578	0.585			
Number of metastatic lymph nodes	1.02	0.977-1.073	0.965			
Number of lymph nodes removed	1	0.980-1.015	0.788			
Lymph node metastasis rate	3.32	1.065-10.382	0.039	3.49	0.93-13.1	0.064
Smoking	1.426	0.713 – 2.850	0.316			
Alcohol	2.531	0.973 – 6.586	0.057			
Hypertension	1.429	0.583 – 3.499	0.435			
Diabetes	0.441	0.121 – 1.610	0.215			

OR, odds ratio; CI, confidence interval; BMI, body mass index; TSH, thyroid-stimulating hormone; FT4, serum free thyroxine; FT3, serum free triiodothyronine; Tg, thyroglobulin; TGAB, thyroglobulin antibody; TPOAB, thyroid peroxidase antibody.

### 3.3 Nomogram prediction of unexpected TSH level

**Figure 1:**
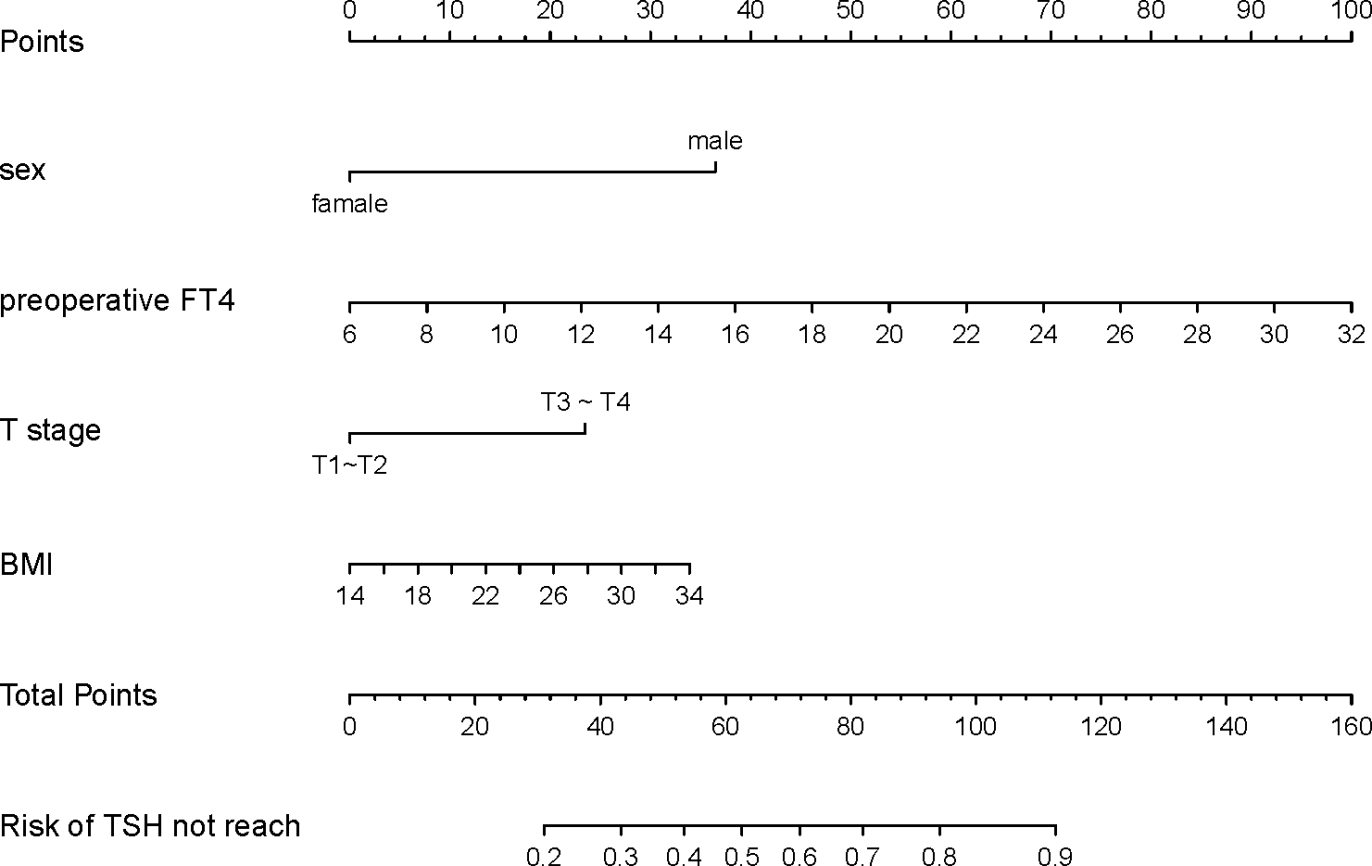
Nomogram developed with sex, preoperative FT4, T stage and BMI incorporated. Assign points to each predictor of the model, reading upwards from the highest score scale at the predictor location to determine the points score related to patient sex, preoperative FT4, T stage, and BMI. Once scores are assigned for each predictor, the total score is calculated. Then, the TSH not reach probability is read through the total points scale. Clinicians can formulate personalized LT4 regimens based on the individual circumstances of the patient and combined with clinical experience.

Based on the final multifactorial logistics regression analysis, a nomogram was developed that incorporates four significant predictors of TSH suppression (**Figure 1**). The total score for sex, BMI, T stage, and preoperative FT4 production served as the basis for this nomogram. Within the nomogram framework, each predictor is represented by a risk point, plotted along the “points” axis. This allows for the calculation of risk points, with the vertical line representing the total score of each predictor’s risk points. When plotted against the “unexpected TSH level” axis, it becomes evident that a higher total score corresponds to an increased difficulty in achieving the objective of TSH suppression.

### 3.4 Performance assessment of the nomogram

**Figure 2:**
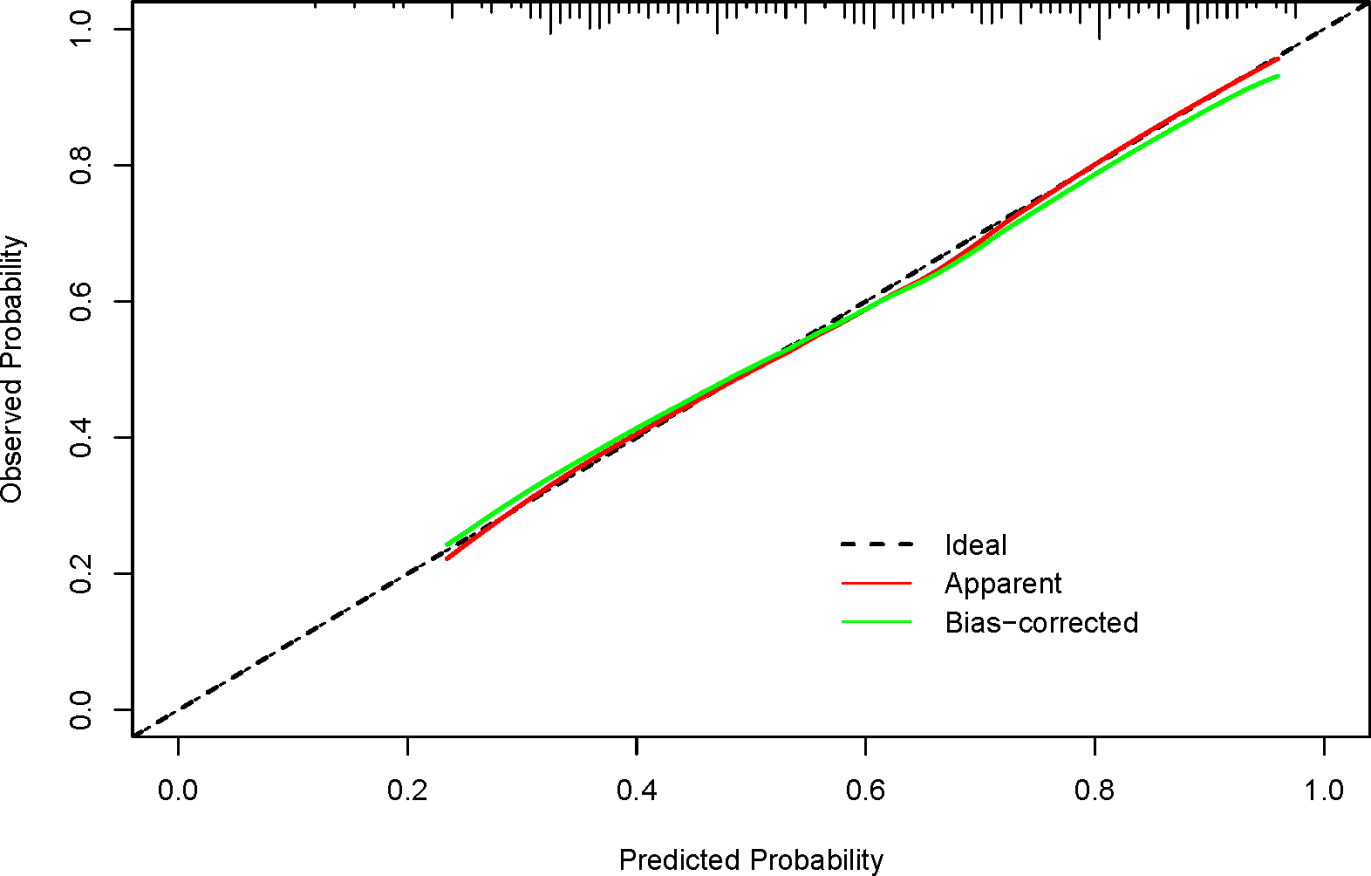
Calibration curves for the nomogram show performance, with the red dotted line representing the entire cohort (n=215) and the green solid line showing bias-corrected results via bootstrapping (500 repetitions). A closer fit of the solid line to the dotted line indicates better prediction. Ideally, the calibration curve should align with the dotted line, reflecting a slope of 1, which signifies high consistency between predicted and actual results. The closer the curve is to this ideal line, the more reliable and better calibrated the model’s predictions are.

**Figure 3:**
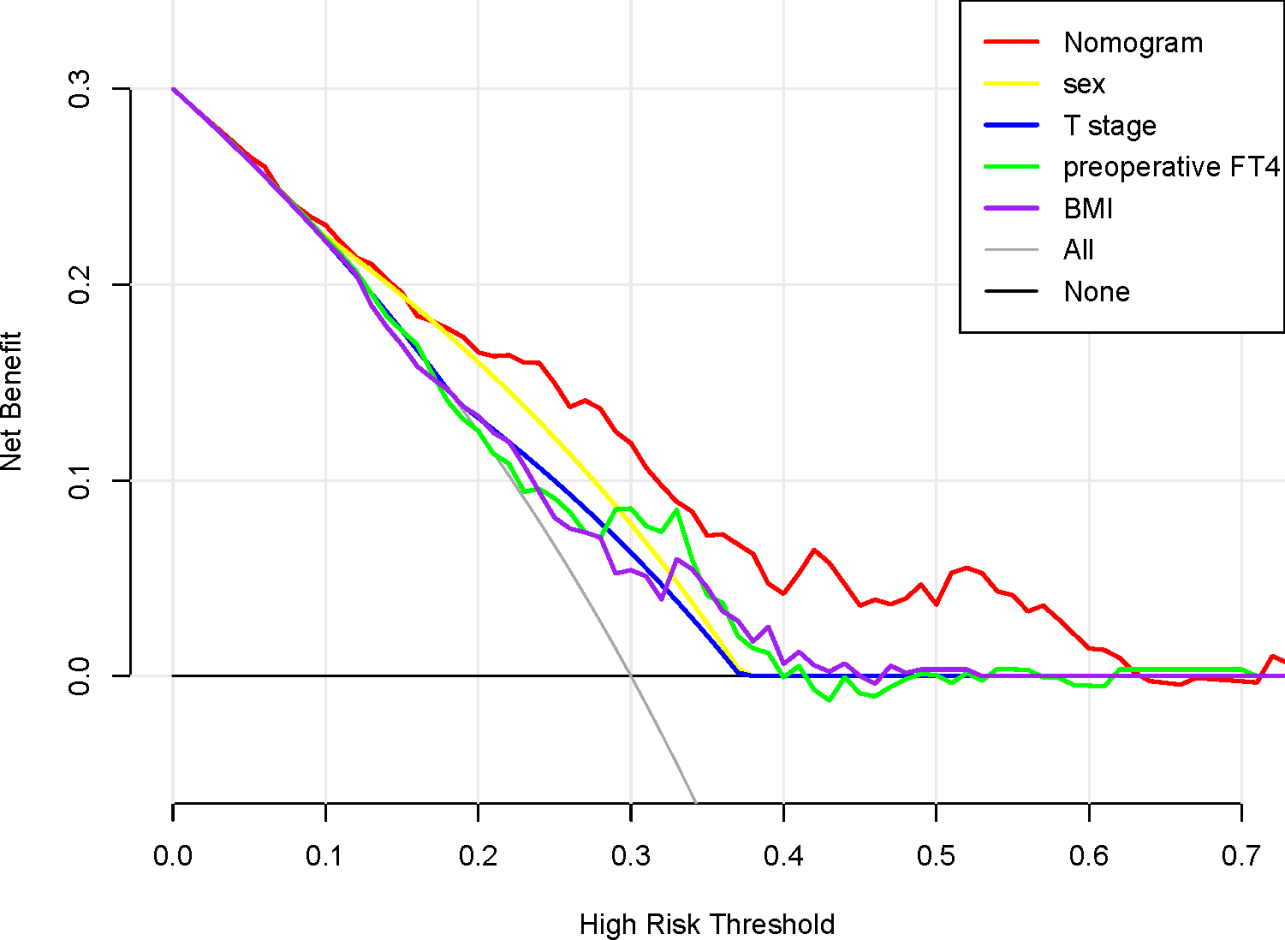
Decision curve analysis for the nomogram, sex, T stage, preoperative FT4 and BMI. The x-axis shows the threshold probability, and the y-axis measures the net benefit. The red line represents the nomogram. The yellow line represents the model with sex. The blue line represents the model with T stage. The green line represents the model with preoperative. The purple line represents the model with body mass index.

We conducted a validation of the nomogram’s performance using a bootstrap method, with 500 repetitions. The nomogram demonstrated moderate discrimination in estimating unexpected TSH levels, as evidenced by the Hosmer-Lemeshow test P = 0.393. The unadjusted C-index was found to be 0.757 (95% CI 0.693–0.822), and the bootstrap corrected C-index was also 0.757 (95% CI 0.687–0.814). These results suggest strong discriminatory power. Furthermore, in cases of TSH suppression, the agreement between the predicted probability and the actual probability, as determined by the calibration curve, was excellent for the nomogram (**Figure 2**). The decision curve analysis (DCA) for both the nomogram plot and the single predictor model are presented in **Figure 3**. When the threshold probability for patients or clinicians falls between 5% and 62%, the predictive effect of the nomogram surpasses that of a single predictor.

## 4 Discussion

Our research found that patient gender, BMI, T stage, and preoperative FT4 level are independent risk factors affecting the achievement of TSH suppression. Patients stratified by different recurrence risks have varying target values for TSH. A simple LT4 dosing regimen based solely on body weight is difficult to achieve satisfactory results (3, 9). Our study analyzed the influencing factors of LT4 medication and has important value in formulating individualized treatment plans.

The lymph node metastasis rate is not affected by the extent of lymphadenectomy and pathological results, and it is a good indicator of tumor load (15). However, compared with other factors (such as T stage, BMI, gender, preoperative FT4 levels), the independent predictive power of possible LT4 demand may be insufficient and did not appear significant in the multiple regression model.

In our study, 60% of patients had TSH levels within the appropriate disease risk range during their initial follow-up. The suppression rate of TSH levels at the first follow-up visit may be a concerning issue, as shown by a study from nine top hospitals in China where, after at least one year of follow-up, 61.4% of patients achieved target TSH levels but with a median time to reach the TSH suppression goal being 234 days (16). In previous studies (12, 17), only 30% of patients achieved the suppressive TSH target at the initial follow-up, possibly due to our higher LT4 initial dose of 2.0μg/kg/day compared to their study’s dose of 1.6μg/kg/day or because our first follow-up time was six weeks post-RAI, which may have allowed for more sufficient decrease in TSH compared to Zhou et al.’s (12) four-week follow-up period.

Gender differences play a crucial role in determining the optimal dosage of LT4. Our study reveals that male patients face challenges in achieving the target TSH level, which aligns with previous research (18). Estrogen levels also influence the required LT4 dosage in women, with premenopausal women requiring higher doses compared to postmenopausal women to attain the desired TSH value (10, 18). However, studies present conflicting findings regarding the necessary LT4 dosage for men. Devdhar (10) proposes on difference between men and postmenopausal women, while Baehr (18) and our study both conclude that men require higher doses than premenopausal women. Additionally, considering physical labor intensity may be essential since Chinese men often engage in more strenuous physical activities. Although the current study adjusted for factors such as age and weight, the effect of gender remains still controversial (19), and further research on the impact of gender on TSH suppression is still very necessary. In future studies, the potential influence of regional differences behind gender should be fully considered, which needs to be confirmed by larger studies.

BMI is an additional determinant influencing TSH suppression, thereby corroborating the perspective of Ojomo et al (9). obese hypothyroid patients often require higher LT4 doses to achieve a normal serum TSH level (10). The impact of body weight on LT4 pharmacokinetics has also been studied. It was found that body weight was a significant predictor of LT4 oral clearance, apparent volume of distribution, and dose-normalized peak concentration (20). Compared with body weight, BMI may serve as a superior indicator. Lean body weight plays a significant role in determining LT4 dosage requirements (10). For overweight individuals with elevated BMI and reduced lean body mass, relying solely on dosing based on body weight can potentially lead to drug overdose. Our study suggests that the challenge faced by high BMI patients in achieving target TSH levels might be partially attributed to excessive medication resulting in lower-than-desired TSH levels.

One intriguing finding is that patients with higher T stages encounter greater challenges in achieving target TSH levels. One potential explanation is that these patients are at a heightened risk of recurrence, leading to lower target TSH values. Additionally, the higher the T stage, the stronger the invasiveness of the tumor, and the patient may need a higher dose of L-T4 to effectively suppress the TSH level (21). Therefore, it is more difficult for them to achieve the desired TSH level within a limited time under the fixed-dose pattern based only on body weight—although our administered dose reached up to 2.0 μg/kg/day. However, our results did not identify the risk of recurrence as an influential factor for LT4 therapy, requiring further research to determine the impact of T stage. Another plausible reason could be that these patients may have undergone more extensive surgical resection, resulting in reduced residual functional thyroid tissue and increased reliance on exogenous thyroid hormones. Therefore, given the same administration method, their ability to meet standard TSH levels becomes more challenging.

Another intriguing finding is that preoperative FT4 levels also play a role in determining LT4 requirements. Zhou et al.’s study (12) revealed that preoperative FT3 is a contributing factor to TSH suppression. Given that both FT4 and FT3 inhibit TSH secretion, their predictive significance may be comparable. In normal individuals, approximately 20% of T3 is produced by the thyroid gland (22); however, thyroid cancer patients lack this hormone due to surgical removal of the thyroid tissue. Consequently, the weight of FT4 in negative feedback of TSH may increase. This viewpoint was supported by Cho et al.’s study (23) which demonstrated elevated levels of FT4 in thyroid cancer patients. Patients with higher preoperative FT4 levels might encounter challenges in achieving TSH suppression due to potential adaptation by the pituitary gland to high levels of FT4. In addition, the FT3/FT4 ratio was found to increase with TSH level in people younger than 40 years, but this phenomenon is not obvious in older people (24). This may imply that the conversion efficiency of thyroid hormone declines with age, thus affecting the demand of LT4.

Our study has several limitations. Firstly, it is a retrospective study conducted at a single center, which may introduce inherent biases and restrict the generalizability. Multicenter cooperative studies should be conducted in the future to improve the representativeness and reliability of the results and reduce selection bias and sampling error. Secondly, our initial dosage was 2.0 μg/kg/day, which is higher than the general dose, potentially limiting the extrapolation of the results. Finally, exercise habits and genetic factors might influence thyroid function and response to LT4 treatment, however their absence in our study limits our ability to assess their impact on the relationships between gender, FT4 levels, T stage, BMI, and TSH levels. This omission may result in an incomplete model and conclusions with limitations. Further research is needed to explore these factors for more personalized protocols. In the future, the nomogram can be validated in different populations. Other factors that may affect LT4 requirements can be explored and added to the prediction model. Prospective studies can also be conducted to evaluate the impact of nomogram-based personalized treatment regimens on patient outcomes.

## Data Availability

The original contributions presented in the study are included in the article/supplementary material, further inquiries can be directed to the corresponding author/s.
